# When transporters fail to be transported: how to rescue folding-deficient SLC6 transporters

**DOI:** 10.29245/2572.942x/2016/9.1098

**Published:** 2016-12-30

**Authors:** Sonja Sucic, Ameya Kasture, H. M. Mazhar Asjad, Carina Kern, Ali El-Kasaby, Michael Freissmuth

**Affiliations:** Institute of Pharmacology, Center of Physiology and Pharmacology, Medical University of Vienna, A-1090 Vienna, Austria

## Abstract

The human dopamine transporter (hDAT) belongs to the solute carrier 6 (SLC6) gene family. Point mutations in hDAT (SLC6A3) have been linked to a syndrome of dopamine transporter deficiency or infantile dystonia/parkinsonism. The mutations impair DAT folding, causing retention of variant DATs in the endoplasmic reticulum and subsequently impair transport activity. The folding trajectory of DAT itself is not understood, though many insights have been gained from studies of folding-deficient mutants of the closely related serotonin transporter (SERT); i.e. their functional rescue by pharmacochaperoning with (nor)ibogaine or heat-shock protein inhibitors. We recently provided a proof-of-principle that folding-deficits in DAT are amenable to rescue *in vitro* and *in vivo*. As a model we used the Drosophila melanogaster DAT mutant dDAT-G108Q, which phenocopies the fumin/sleepless DAT-knockout. Treatment with noribogaine and/or HSP70 inhibitor pifithrin-μ restored folding of, and dopamine transport by, dDAT-G108Q, its axonal delivery and normal sleep time in mutant flies. The possibility of functional rescue of misfolded DATs in living flies by pharmacochaperoning grants new therapeutic prospects in the remedy of folding diseases, not only in hDAT, but also in other SLC6 transporters, in particular mutants of the creatine transporter-1, which give rise to X-linked mental retardation.

Point mutations in a gene, which result in misfolding of the encoded protein, are known to be the underlying cause of many diseases. In fact, the term molecular medicine was coined in conjunction with the study of sickle cell anaemia, the prototypical protein folding disease^1^. With a few prevalent exceptions (e.g. sickle cell anaemia), individual folding diseases are rare (i.e., affecting less than 1 in 2000 persons), but collectively they affect many people. In addition and more importantly, it has been pointed out more than 75 years ago that the study of rare diseases is intrinsically worthwhile: major scientific advances have been achieved by studying rare diseases^2^. We posit that this also true for the study of misfolded versions of neurotransmitter transporters of the SL6 family. The human genome encodes twenty members of the solute carrier 6 (SLC6) gene family, but SLC6A10 (creatine transporter-2/CT-2) is a pseudogene: SLC6 transporters can be grouped into 4 families based on their evolutionary relation, namely (i) the monoamine transporters for dopamine (DAT, SLC6A3), serotonin (SERT, SLC6A4) and noradrenaline (NET, SLC6A2), (ii) the amino acid/neurotransmitter transporters (e.g. glycine tranporters-1 and -2 = SLC6A9 and SLC6A5, respectively), the GABA/osmolyte-transporters (including GABA-transporters GAT-1 to -4 = SLC6A1, SLC6A13, SLCA11 & SLCA12) and (iv) the nutrient/orphan amino acid transporters^3^. SLC6 transporters operate as NaCl^-^dependent transporters; they exploit the electrochemical gradient for Na^+^ to drive inward transport of substrate in a conformational cycle comprising an outward-facing conformation, a substrate- and Na^+^- and Cl^-^-bound (stoichiometry 2 or 3 Na^+^, 1 Cl^-^) occluded state, an inward-facing conformation and a K^+-^ and Cl^-^-bound return step^4^. In addition, SLC6 transporters can also operate in the substrate-exchange mode, which is the basis for amphetamine-induced reverse transport by DAT, NET and SERT; this accounts for the psychostimulant actions of amphetamines^5^.

Missense mutations, which give rise to a human disease, occur in many SLC6 transporters^6^. In three instances, these mutations have been shown to cause misfolding of the mutated SLC6 transporter, i.e. in NET, DAT and GlyT2^6^. A single mutation in NET (A457P) causes postural hypotension/orthostatic intolerance, which is genetically transmitted in a dominant fashion^7^. There are more than 15 mutations in DAT, which result in a folding defect and cause a recessive form of infantile/juvenile dystonia/Parkinson’s disease^8^–^10^ and at least 8 mutations, which result in misfolding of GlyT2 and thus lead to hyperekplexia/startle disease^11^–^14^. Two of these mutations act in dominant manner (see below). Finally, there is one SLC6 transporter, i.e. the creatine transporter-1 (CT-1/SLC6A8), where the circumstantial evidence suggests that - at least in some of the 22 missense mutations^15^ - the disorder (mental retardation) is also due to misfolding of the mutated transporter (see below). SLC6 transporters epitomise the folding problem of dynamic polytopic membrane proteins: SLC6 transporters have 12 transmembrane spanning (mostly α-helical) segments; thus the bulk of the protein is hydrophobic in nature. If all known mutations of SLC6 transporters, which are known or suspected to cause misfolding, are mapped onto a structural model of SLC6 transporters (based on the available crystal structures of DAT), they are found to be enriched at the lipid/protein-interface^6^. This indicates that the lipid bilayer imposes a major constraint as the nascent protein moves through the conformational search space to reach a stable fold. This can be rationalized, if the individual steps of the folding trajectory are recapitulated (for details see ref. 6): (i) the transmembrane helices are co-translationally inserted into the SEC61 translocon of the endoplasmic reticulum. At this stage, the motion of amino acid side chains is restricted, thus limiting the search space. (ii) Transmembrane segments are released individually or in pairs of two via a lateral gate of SEC61 into the lipid bilayer^16^ (Figure 1a). (iii) Within the lipid bilayer, the transmembrane segments of all polytopic membrane proteins have to rearrange, because they typically adopt an annular rather than a serpentine topology. In nascent SLC6 transporters, this requires lipids to be displaced from those surfaces of the 12 α-helical transmembrane segments, which face each other or from the translocation pathway (Figure 1b). The annular arrangement must be bolted to reach the stable fold. (iv) Finally, it is worth considering that SLC6 transporters have - by definition - a flexible conformation, because they must support the transport cycle (see above). The question thus arises whether the folding trajectory proceeds to the outward or the inward facing conformation.

## A keyhole perspective of the folding problem

It is obvious that the folding trajectory of SLC6 transporters must move through a conformational search space but the underlying details are not known. However, several serendipitous insights offer a glimpse of the problem and allow for generating testable models. In addition, the SLC6 folding mutants, which cause human diseases, provide a backdrop to examine the models for their explanatory power. The first relevant finding is the observation that SLC6 transporters form constitutive oligomers^17^. The oligomers are kinetically trapped at the cell surface, i.e. they do not exchange^18^ but not within the endoplasmic reticulum (ER)^19^. Thus, oligomerization occurs in the ER. Oligomerization in the ER is a prerequisite for ER-export^20^; conversely, export-deficient versions of GAT1 or of SERT exert a dominant-negative effect on the wild type protein^21^,^22^. Thus, based on these findings it is possible to rationalize the dominant negative effect of misfolded SL6 transporters such as NET-A457P^7^, GlyT2-S510R and related mutants^11^,^13^,^14^.

**Figure 1:**
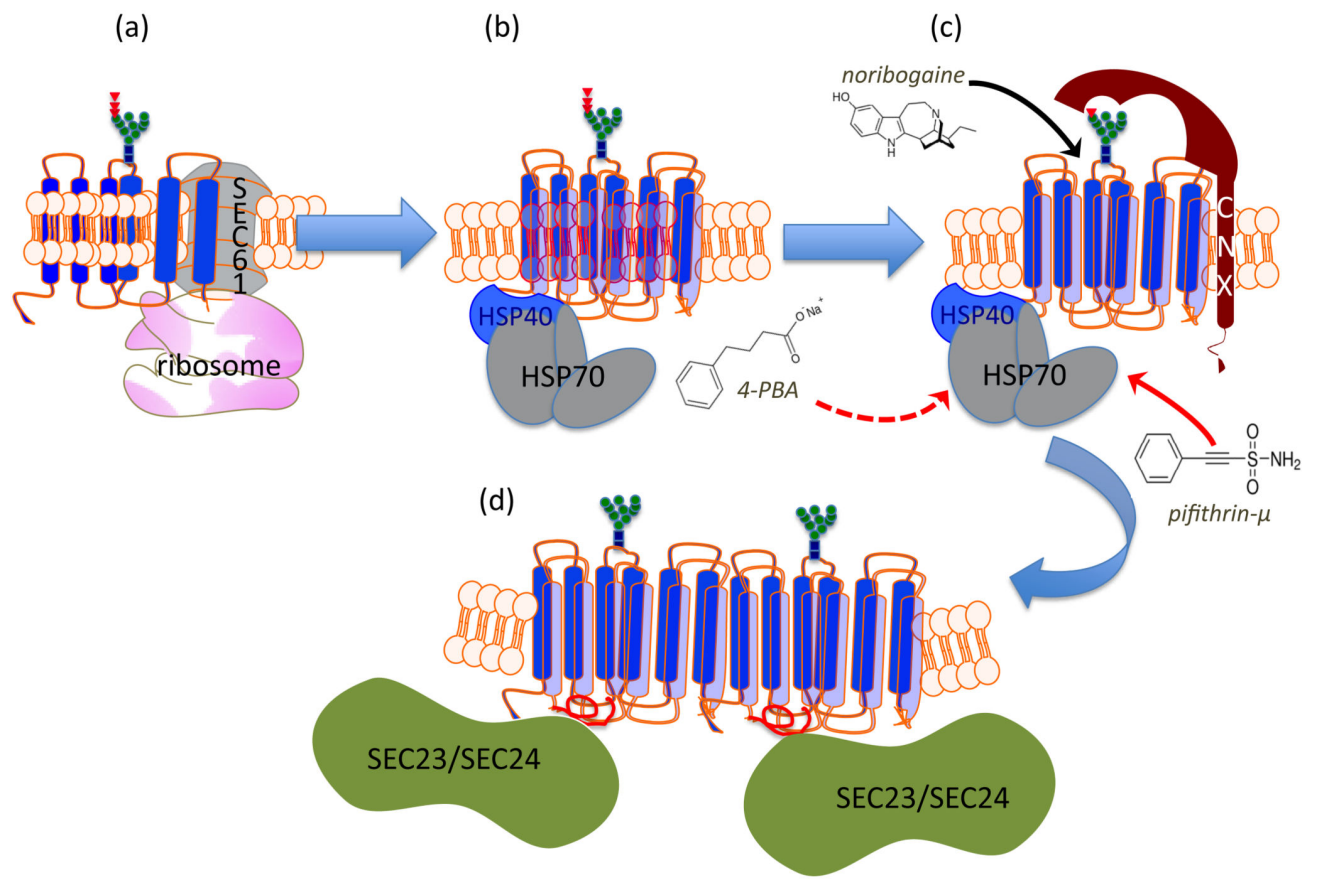
Schematic representation of the sites of action of pharmacochaperones and chemical chaperones in the folding trajectory of DAT. (a) The first transmembrane segment of nascent DAT acts as a signal peptide. The ribosome is recruited to the membrane of the endoplasmic reticulum (ER) via the signal recognition particle (SRP) and the SRP receptor (not shown), where the translation arrest is lifted and the transmembrane helices are cotranslationally inserted into the translocon/SEC61 channel. The helices are released into the ER membrane via a lateral gate. On the lumenal side (topologically equivalent to the extracellular side), the nascent protein chain is subject to N-linked core glycosylation (blue squares represent N-actely-glucosamin, green dots mannose and triangles glucose). (b) To achieve an annular arrangement, lipids have to be expelled from the interior of the ring. On the cytosolic side, the C-terminus is engaged by a heat-shock protein relay (shown here is a dimer of HSP40 and HSP70) to promote folding and to preclude premature engagement of the COPII-coat. (c) During folding, ER-resident, lumenal chaperones are recruited to folding intermediates: shown here is calnexin (CNX), which recognizes the (re)glucosylated folding intermediates via its lectin domain. (d) When the minimum energy conformation - i.e. the stably folded state - is reached the chaperones are released: the transporter forms an oligomer and the cognate SEC23/SEC24-dimer (containing SEC24D for DAT, see ref. 39) is recruited to the C-terminus, which contains an α-helix (highlighted in red). This C-terminal α-helix interacts with the first intracellular loop and thus bolts the annular arrangement of hydrophobic core. The bow-tie shape of the COPII-component SEC23/SEC24 stabilizes the membrane curvature of the nascent vesicle, which will carry the transporter en route to the Golgi. Noribogaine binds to the ligand/substrate binding-site within the hydrophobic core and stabilizes the inward facing conformation. This lowers the energy barrier between folding intermediates and thus facilitates the progression along the folding trajectory. Pifithrine-µ inhibits HSP70 and is thought to thereby release stalled transporter complexes. 4-Phenylbutyric acid (4-PBA) modulates the expression levels of various HSP70 family members. This is thought to shift the balance in favor of progression through the folding trajectory, while the formation of stalled complexes is reduced.

However, this doesnot explain the recessive transmission of the other hyperekplexia-causing mutations in GlyT2^11^,^12^ or all known mutations in DAT, which give rise to childhood dystonia/parkinsonism^8^–^10^.The apparent oxymoron of having both, dominant and recessive SLC6 mutations, can be resolved by considering that oligomer formation occurs late in the folding trajectory. In fact, the available evidence supports a model, where the nascent SLC6 transporter is engaged by several chaperones in the ER lumen, most notably calnexin^23^ and a cytosolic chaperone relay, which engages the C-terminus of the transporter^24^ (Figure 1b and Figure 1c). Based on this model^6^, it is possible to rationalize how a recessive transmission operates: the misfolded SLC6 transporter is trapped by ER-resident lumenal chaperones (e.g. calnexin), which precludes oligomer formation with the product of the wild type allele.

One way to approach the folding problem is to work backwards from the folded state and ask, which conformation(s) was/were visited before the folded state was reached. A serendipitous finding was the observation that the drug noribogaine corrected the folding deficit of several SERT mutants, which had been created to study ER export^25^ and the folding problem^24^. Ibogaine and its derivative noribogaine bind to and stabilize the inward facing conformation^26^,^27^. This implies that the folding trajectory proceeds through the inward facing conformation. Accordingly, mutations, which trap the transporter in the inward facing state, are predicted to remedy the folding deficit. Several inward-facing mutant versions of SERT are available^28^,^29^. If these mutations are introduced into folding-deficient mutants of SERT, they act as second site suppressors, i.e. they restore folding and promote ER export of the double mutants, although the extent to which individual mutants are rescued differs^30^. Thus, the available evidence suggests that the folding trajectory moves through the inward facing conformation.

As mentioned above, the annular arrangement of the transmembrane helices must be stabilized during the folding trajectory. It was appreciated more than a decade ago that serial truncation of GAT1^31^ or of SERT^32^ impaired surface expression. Similarly, mutations within the first intracellular loop of NET also affect the delivery of the transporter to the plasma membrane^33^–^35^. These two sets of information can be rationalized: both, the first intracellular loop and the C-terminus are required for folding, because a salt bridge is formed between the end of a helical segment in the C-terminus and the first intracellular loop; this interaction presumably stabilizes the annular arrangement of the hydrophobic core and thus facilitates folding of SERT^30^. The C-terminus of SERT is shielded by a heat-shock protein relay^24^: folding-deficient mutants are stalled in different complexes. This association provides a handle to assess progression of SERT and - by inference - of other SLC6 transporters through the folding trajectory^24^,^30^. In addition and importantly, this insight allows for targeting the folding machinery with drugs (Figure 1; see also below).

## The acid test - rescuing mutated transporters

**Figure 2:**
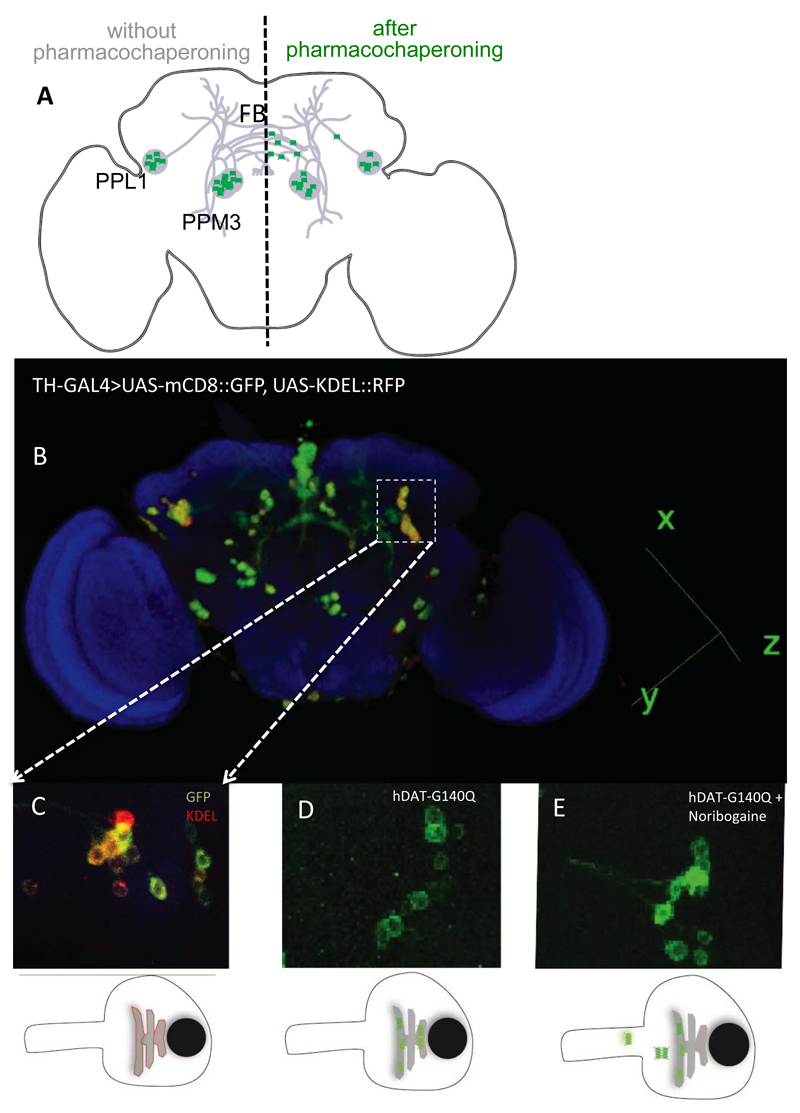
Pharmacochaperoning rescues a misfolded human DAT (hDAT-G104Q) in the brain of Drosophila melanogaster. A) Schematic cartoon showing the dopaminergic dorsomedial posterior protocerebral (PPM3) and dorsolateral posterior protocerebral neurons (PPL1) neurons, which project their axons into the fan-shaped body (FB) of the fly brain. In the absence of pharmacochaperoning (left hand side) the mutant DAT mutant (green dots) is retained in the ER; if flies are administered noribogaine via their food, a considerable fraction of the DAT mutant reaches the presynaptic specialization (right hand side). B) Posterior view of 3D rendered adult fly brain expressing the surface marker mCD8-green fluorescence protein (GFP) and the ER marker red fluorescence protein (RFP)-KDEL under the control of tyrosine hydroxylase GAL4 (TH-GAL4). C) Magnified image of paired posterior lateral 1 (PPL1) cluster of dopaminergic neurons. D and E. TH-GAL4 driven expression of hDAT-G140Q in PPL1 neurons in the brain of untreated flies (D) and flies receiving noribogaine (100 μM) in their food (E). It is evident from panel E that the GFP-tagged (=green) hDAT-G140Q entered the axonal extension, whereas in panel D it is confined to the ER in the cell soma. The bottom panels represent schematic cartoons of the fluorescent images shown above outlining the red fluorescence of KDEL in the ER within the soma (left), the green fluorescence of the DAT mutant in untreated flies (middle), which upon pharmacochaperoning leaves the ER and enters into neurites (right).

The vast majority of missense mutations in human DAT, which cause childhood dystonia/parkinsonism, give rise to a folding-deficient transporter, which is retained in the ER^8^-^10^. The phenotypic consequence is that of dopamine deficiency rather than a hyperdopaminergic state, because vesicular stores of dopamine are not replenished. The acid test for any models, which summarize the rudimentary understanding of SLC6 transporter folding, is their ability to guide attempts to rescue folding-deficient transporter mutants. We translated the insights, which we had gained by studying ER export of GAT1^20^,^21^,^31^,^36^,^37^, and SERT^23^,^38^–^40^ and the rescue of folding deficient mutants^24^–^25^,^30^, to a DAT mutant, which was serendipitously discovered in Drosophila melanogaster: flies harboring Drosophila DAT-G108Q (dDAT-G108Q) have very much reduced sleep time^41^. Hence this mutation phenocopies the DAT knockout in flies (referred to as fumin, i.e. sleepless)^42^. Glycine 108 is part of a GXXXG-related motif, which stabilizes the interaction between TM3 and TM12 at the intracellular leaflet of the membrane. When substituted by the large glutamine, the mutation interferes with packing of helix TM12 in the folding trajectory of dDAT. This in turn affects the C-terminus, which closely follows TM12^43^. As outlined above, the C-terminus is positioned to stabilize the annular arrangement of the hydrophobic core. Accordingly, in transfected cells, dDAT-G108Q and its human equivalent hDAT-G140Q were trapped in the ER in complex with calnexin and HSP70-1A, reflecting the stalling of the mutant along the folding trajectory^43^. The folding defect of dDAT-G108Q was remedied by the pharmacochaperone noribogaine or the HSP70 inhibitor pifithrin-μ:, the mutant reached the cell surface, and transport activity was also recovered. Most importantly, this pharmacochaperone action observed in cell cultures was reproduced *in vivo* in dDAT-G108Q flies: upon pharmacochaperone treatment the mutant reached the axonal projections (Figure 2) to a level sufficient to recover sleep^43^. Axonal targeting is obviously important, because the refilling of vesicular stores of neurotransmitters depends on this eponymous action of DAT: mutants of GAT1 and SERT, which fail to recruit their cognate SEC24-isoform eventually do reach the cell surface, but they are not delivered to the presynaptic specialization^36^,^37^,^40^. Thus, the fact that dDAT-G108Q and hDAT-G140Q reached the axonal territory shows that neither pharmacochaperoning by noribogaine nor inhibition of HSP70 by pifithrin-µ reroute the transporter through an atypical ER export pathway. Based on these findings, it was sound to predict that some of the misfolded mutants of DAT ought to be rescued by pharmacochaperoning with noribogaine, and that childhood dystonia/parkinsonism may be amenable to treatment by pharmacochaperones and/or HSP70 inhibition, which restore folding of the mutated DAT-versions. In fact, some aspects of this prediction have already been verified in transfected cells: cell surface expression of several disease-causing DAT-mutants was restored by pharmacochaperoning with ibogaine and with bupropion^44^, which is of particular interest, because bupropion is an approved drug.

The observations on dDAT-G108Q also have repercussions for mutations in creatine transporter-1 (SLC6A8): a mutation of the equivalent glycine (G132V) is found in boys with mental retardation^15^,^45^. It is therefore reasonable to assume that CT1-G132V is also misfolded and that is also amenable to rescue by pharmacochaperoning and/or inhibition of heat-shock proteins. The monoamine transporters DAT, SERT and NET have a rich pharmacology^5^: several hundred inhibitors and substrate analogues are available, which is a treasure trove in the search for pharmacochaperones. In contrast, the number of CT1-ligands is limited. This makes the inhibition of heat-shock proteins or the manipulation of their expression by 4-phenylbutyrate^46^,^47^ of particular interest to restore folding and surface expression of mutated versions of CT1.
